# Terminal Restriction Fragment Length Polymorphism Analysis of Soil Bacterial Communities under Different Vegetation Types in Subtropical Area

**DOI:** 10.1371/journal.pone.0129397

**Published:** 2015-06-22

**Authors:** Zeyan Wu, Wenxiong Lin, Bailian Li, Linkun Wu, Changxun Fang, Zhixing Zhang

**Affiliations:** 1 Life Sciences College of Fujian Agriculture and Forestry University, Fujian, China; 2 Ecological Complexity and Modeling Laboratory, University of California Riverside, Riverside, CA, United States of America; Shanxi University, CHINA

## Abstract

Soil microbes are active players in energy flow and material exchange of the forest ecosystems, but the research on the relationship between the microbial diversity and the vegetation types is less conducted, especially in the subtropical area of China. In this present study, the rhizosphere soils of evergreen broad-leaf forest (EBF), coniferous forest (CF), subalpine dwarf forest (SDF) and alpine meadow (AM) were chosen as test sites. Terminal-restriction fragment length polymorphisms (T-RFLP) analysis was used to detect the composition and diversity of soil bacterial communities under different vegetation types in the National Natural Reserve of Wuyi Mountains. Our results revealed distinct differences in soil microbial composition under different vegetation types. Total 73 microbes were identified in soil samples of the four vegetation types, and 56, 49, 46 and 36 clones were obtained from the soils of EBF, CF, SDF and AM, respectively, and subsequently sequenced. The *Actinobacteria*, *Fusobacterium*, *Bacteroidetes* and *Proteobacteria* were the most predominant in all soil samples. The order of Shannon-Wiener index (H) of all soil samples was in the order of EBF>CF>SDF>AM, whereas bacterial species richness as estimated by four restriction enzymes indicated no significant difference. Principal component analysis (PCA) revealed that the soil bacterial communities’ structures of EBF, CF, SDF and AM were clearly separated along the first and second principal components, which explained 62.17% and 31.58% of the total variance, respectively. The soil physical-chemical properties such as total organic carbon (TOC), total nitrogen (TN), total phosphorus (TP) and total potassium (TK) were positively correlated with the diversity of bacterial communities.

## Introduction

It is well known that the interaction between plants and soil microbes is one of the forefront topics of international ecological research [1, 2]. Soil microbial characteristics have been studied intensively in recent two decades since soil microorganisms play a critical role in energy flow and material exchange of the forest ecosystems [3, 4]. Many factors, such as temperature [5], water content [6], pH [7], soil type [8], and soil depth [9], influence soil microbial communities. The effects of vegetation types on soil microorganisms have also been reported in a number of studies. For example, consistent difference in microbial communities were observed among crop species by using phospholipid fatty acidfrom (PLFA) from soil microbial communities [10]. Hack *et al*. discovered that specific soil conditions under different forest types embraced specific soil microbial communities [11]. Vegetation types also play an important role in structuring soil bacterial communities [12]. Weand *et al*. further reported that both the composition of microbial community and enzyme activities varied with different vegetation types [13].

A deeper understanding about the effects of the vegetation types on the composition of the soil microbial communities in a forest ecosystem can help devise better strategies and management practices to improve soil health and plant growth [14]. However, limited information is currently available on how the vegetation types influence the composition and diversity of soil microbial communities in Wuyi Mountains. The Wuyi Mountains is located in the southeast of China, and have distinct vertical zonation of different vegetation types and rich plant resources. It is a typical representation of the subtropical forest ecological system, and provides a unique opportunity to study the plant-soil microbial interactions [15, 16].

The diversity of microorganisms in soil is very abundant; however, traditional microbial culture method has its limitations to study the composition of microbial communities because only 1% of the soil microorganisms present can be cultured [1, 17]. Terminal restriction fragment length polymorphism (T-RFLP) is a culture-independent technique and has higher resolution and more comprehensive than cultivation-based methods [18, 19]. In recent years, this technique has been successfully applied to the composition and diversity analysis of soil microbial communities under different environmental conditions [4, 20, 21, 22]. In this study, T-RFLP analysis method was used to reveal the composition and diversity of soil bacterial communities along an altitude gradient in Wuyi Mountains. Our purpose was to address two questions: (1) is there a relationship between the composition and structure of the soil microbial communities and the vegetation types? (2) What causes the differences of soil microbial communities’ properties under different vegetation types?

## Materials and Methods

### Ethics statement

This study has been approved by the Wuyi Mountain National Nature Reserve Management Committee, which takes care of the planning and protecting of Wuyi Mountain. The study did not involve any endangered or protected species. All the data in this study can be published and shared.

### 2.1 Sampling sites

The Wuyi Mountain National Natural Reserve has the largest subtropical primordial forest ecosystem at the same altitude of the world, and is one of the key regions of biodiversity protection worldwide. It is located in Fujian Province (27°32′-27°55′ N, 117°24′-118°02′E), a 565 km^2^ forested area in the subtropical monsoon region of China [23]. The annual average temperature is 17.6°C and the annual precipitation is 1864 mm with an annual mean relative humidity of 83.2% [23]. The annual average frost-free period is about 102 days. Huanggang Moutain is the pulse peak and 2158 meters above sea level. Four typical vegetation types are distributed along the altitude gradient: evergreen broad-leaf forest (EBF), coniferous forest (CF), sub-alpine dwarf forest (SDF), and alpine meadow (AM) [24]. The specific conditions of the four sampling sites are shown in Table 1.

**Table 1:** Description of the four vegetation types along an altitude gradient in the Wuyi Mountains.

Vegetation types	Altitude (m)	Annual mean temperature (°C)	Annual mean precipitation (mm)	Soil types	Dominant species
EBF	500	18.3	1751	Red earth	*Castanopsis carlesii*
CF	1150	14.7	2014	Yellow earth	*Pinus tanwanensis*
SDF	1750	11.6	2167	Yellow earth	*Symplocos paniculata*; *Stewartia sinensis*
AM	2150	9.8	3058	Mountain meadow soil	*Calamagrostis brachytricha*; *Miscanthus sinensis*; *Lycopodium clavatum*

### 2.2 Sample collection and analysis of soil physical-chemical properties

Three replicate soil sampling plots (20 m×20 m) were selected in each site along an altitude gradient in August 2013. Soil samples were randomly collected from 0–20 cm soil depths in each plot using a soil core sampler (diameter of 2.0 cm). Twenty cores were composited into one soil sample, which were then sieved (2 mm) to remove soil impurities, hand-mixed and stored in plastic bags. Half of each soil sample was stored at 4°C until analysis, and another half was air-dried and sieved to determine soil pH, moisture content, total organic carbon (TOC), total nitrogen (TN), total phosphorus (TP) and total potassium (TK) as described by Wu *et al*. [25].

### 2.3 Analysis of terminal restriction fragment length polymorphism (T-RFLP)

#### 2.3.1 Nucleic acid amplification and restriction digestion

We used the high salt/SDS method to extract the DNA from soil samples as reported previously [26]. The amounts of the extracted DNA were detected using a BioPhotometer (Eppendorf, Hamburg, Germany) and the size of the DNA was checked on a 0.8% agarose gel. Extracted DNA was stored at -20°C until analysis. One pair of primers, 8-27F and 926-907R, were used to amplify the 16S rRNA genes by PCR. Four restriction enzymes *Msp*I, *Hae*III, *Rsa*I and *Alu*I were used to digest the purified 16S rRNA fragments, followed the method as described by Nithya & Pandian [18]. Samples denatured at 96°C for 4 min, chilled on ice and then run in an automated ABI DNA sequencer [19].

#### 2.3.2 Analysis of T-RF profiles

The GeneMarker (Version 1.2) was used to analyze the T-FRs. Fragments smaller than 50 or larger than 1200 bp were deleted from the analysis. The relative abundance of each T-RF was calculated as the peak area of the respective T-RF divided by the total peak area of all T-RFs [19]. Affiliations of the fragments were determined via online T-RFLP analysis of Ribosomal Database Project II [19]. The statistical analysis of was done on the basis of complete sample profiles. Several diversity indexes such as Species Richness index (*S*), Shannon-Weiner index (*H*), Pielou’s evenness index (*E*) and Simpson index (*D*) were calculated as described previously in order to measure species diversity of bacterial communities [16]. The correlation analysis was used to analyze the relationship between bacterial community diversity and soil physicochemical properties. Principal components analysis (PCA) was performed to compare the T-RFLP profiles of different samples followed the method as described by Park *et al*.[1]. To test significant differences between the sampling sites, we performed one way analysis of variance (ANOVA) followed by Tukey's tests (*P* < 0.05) through SPSS 17.0.

## Results

### 3.1 Comparison of bacterial communities’ composition among distinct vegetation types

**Fig 1:**
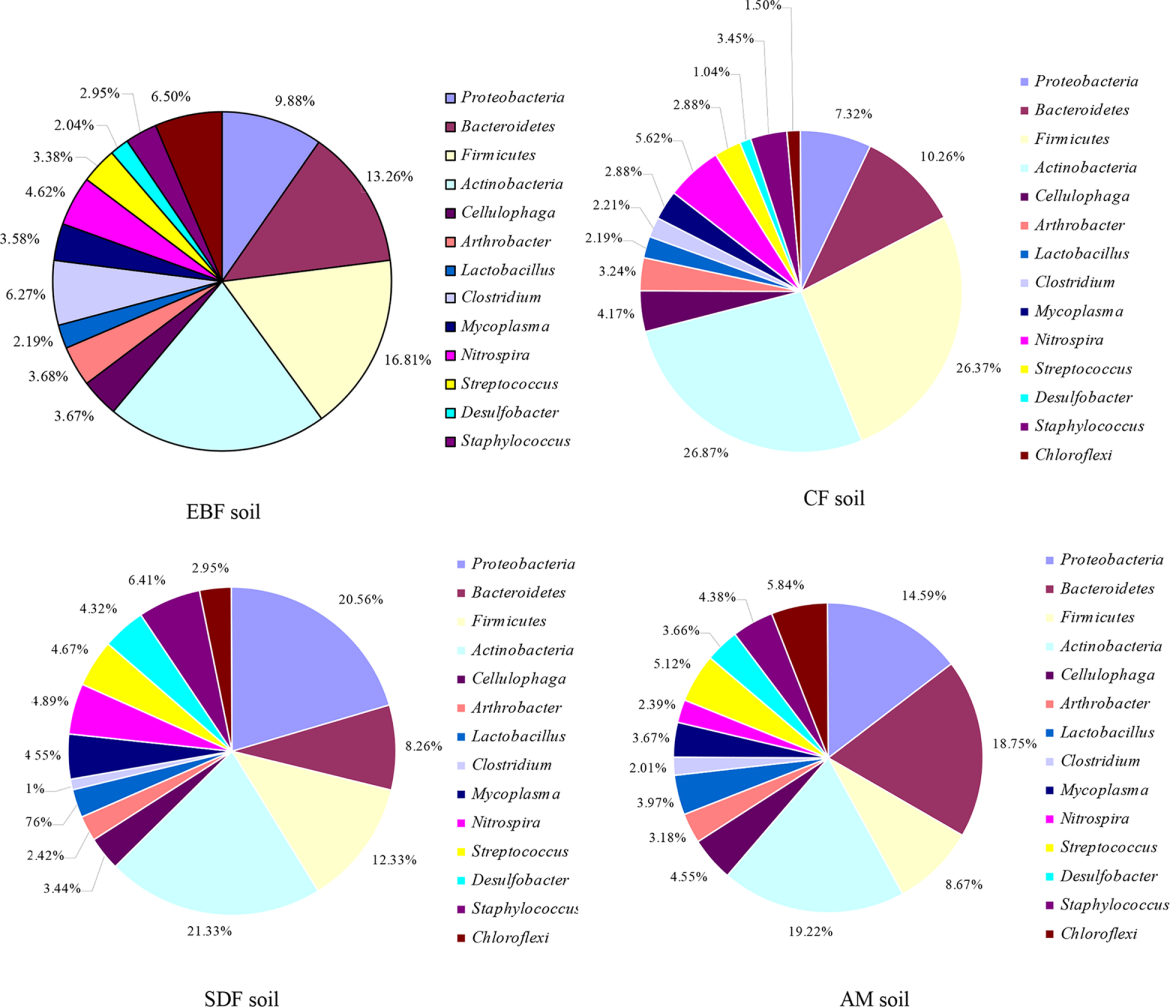
Schematic representation of bacterial communities in soil samples of different vegetation types. The figure shows the differences of bacterial communities’ composition among different vegetation types (EBF, CF, SDF and AM).

All the experimental data of T-RFLP were listed in Supporting Information (S1 File). According to Table A in S1 File, total 73 microbes were identified in soil samples of the four different vegetation types, which 56, 49, 46 and 36 clones were obtained and sequenced from the soil samples of EBF, CF, SDF and AM, respectively (Table B, Table C, Fig. A in S1 File). All microbes can be categorized into 14 phylum, 21 class and 66 species. 14 phylum were *Proteobacteria*, *Bacteroidetes*, *Fusobacterium*, *Actinobacteria*, *Cellulophaga*, *Arthrobacter*, *Lactobacillus*, *Clostridium*, *Mycoplasma*, *Nitrospira*, *Streptococcus*, *Desulfobacter*, *Staphylococcus* and *Chloroflexi*. The *Actinobacteria*, *Fusobacterium*, *Bacteroidetes* and *Proteobacteria* were four dominant phylum in all soil samples (Fig 1, Table D in S1 File). For EBF samples, two of the predominant T-RFs were affiliated to *Acidobacteria* (21.37%) and *Fusobacterium* (16.81%), whereas *Acidobacteria* (26.87%) and *Fusobacterium* (26.37%) were also the most dominant in CF. Compared with the clone sequences retrieved from EBF and CF samples, the predominant T-RFs were affiliated to *Acidobacteria* (21.33%) and *Proteobacteria* (20.56%) in SDF samples, whereas *Acidobacteria* (19.22%) and *Bacteroidetes* (18.75%) were found in AM samples.

### 3.2 Diversity analysis of soil bacterial communities among distinct vegetation types

Four diversity indexes obtained from different restriction enzymes were showed in Table 2. For the species richness at EBF samples, the number of T-RFs (32) obtained with *Alu* I was 37.25% lower than those (51) with *Hae* III. A similar tendency of variation was also found in the CF, SDF and AM samples. The Species Richness index (*S*), Shannon-Weiner index (*H*), Pielou’s evenness index (*E*) and Simpson index (*D*) were then used to show the species diversity of bacterial communities. Correlation coefficients among species indices in the bacterial communities showed that the Pielou’s evenness index had no correlation with other diversity indexes. The Shannon-Weiner index was significantly correlated with Species Richness index and Simpson index, and it could be regarded as the key indicator of species diversity of the bacterial communities (Table 3). Results from all four restriction enzymes suggest that the Shannon-Weiner index in the soil of different vegetation types followed the order of EBF>CF>SDF>AM by using *Hae* III, *Msp* I and *Rsa* I, but followed the order of EBF>SDF>CF>AM when *Alu* I was used. No matter which restriction enzymes we used, the Shannon-Weiner index of EBF showed maximum richness and AM showed minimum richness, indicating that the diversity of soil bacterial communities decreased with increasing elevation, and revealed a general trend of EFB>CF>SDF>AM.

**Table 2:** Diversity indexes obtained from different restriction enzymes in soil samples of different vegetation types.

Vegetation types	Diversity indexes															
	Species Richness (*S*)				Shannon-Weiner index (*H*)				Pielou’s evenness index (*E*)				Simpson index (*D*)			
	*Hae*III	*Msp* I	*Rsa* I	*Alu* I	*Hae* III	*Msp* I	*Rsa* I	*Alu* I	*Hae* III	*Msp* I	*Rsa* I	*Alu* I	*Hae* III	*Msp* I	*Rsa* I	*Alu* I
EBF	51	48	31	32	3.895	2.589	2.833	2.673	0.876	0.831	0.914	0.873	0.966	0.957	0.974	0.925
CF	47	44	41	30	3.531	2.302	2.593	1.797	0.901	0.886	0.895	0.849	0.942	0.949	0.938	0.906
SDF	38	33	40	29	3.290	2.244	2.486	1.809	0.925	0.908	0.838	0.852	0.873	0.906	0.882	0.804
AM	36	29	30	24	2.784	2.012	2.019	1.572	0.822	0.850	0.867	0.817	0.799	0.833	0.801	0.765

**Table 3:** Correlation coefficients among species indices for the bacterial communities.

	*Hae* III				*Msp* I				*Rsa* I				*Alu* I			
	*S*	*H*	*D*	*E*	*S*	*H*	*D*	*E*	*S*	*H*	*D*	*E*	*S*	*H*	*D*	*E*
*S*	1				1				1				1			
*H*	0.966**	1			0.985**	1			0.941**	1			0.959**	1		
*D*	0.903**	0.844*	1		0.947**	0.819*	1		0.933**	0.877*	1		0.924	0.865*	1	
*E*	0.105	0.258	0.009	1	0.206	0.193	0.174	1	0.161	0.310	0.275	1	0.211	0.148	0.096	1

Note:

*means *P* < 0. 05, significant correlation,

**means *P* < 0. 01, extremely significant correlation in this and subsequent tables.

**Fig 2:**
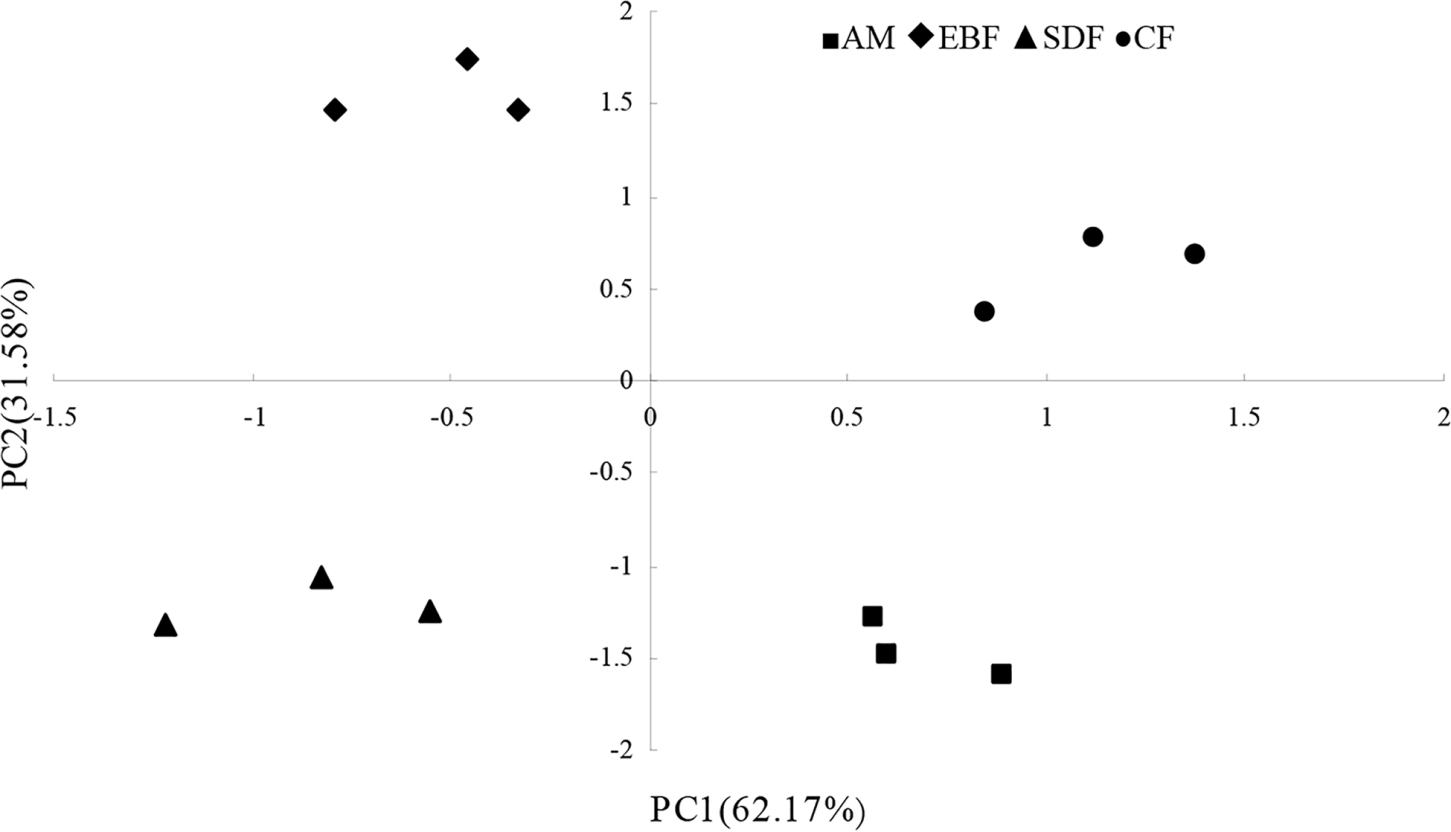
Principal components analysis (PCA) of T-RFLP data in different vegetation types along an altitude gradient. The figure describes the variance of bacterial communities in the EBF, CF, SDF and AM sites were clearly different from each other.

Principal components analysis (PCA) of T-RFLP data in different vegetation types along an altitude gradient was showed in Fig 2 (Table E in S1 File). The PCA score plot of T-RF data revealed that the structures of soil bacterial communities in the EBF, CF, SDF and AM sites were clearly different from each other, with EBF and SDF on the left side, and CF and AM on the right side of the axis, which described 62.17% and 31.58% of the total variance, respectively.

### 3.3 Relationship between soil physical-chemical properties and bacterial communities’ composition

Table 4 shows the results of physical-chemical analysis for the soil samples collected from the four different vegetation types. The soil properties selected for this study were significantly different among the different study sites (*P*<0.05). The soil pH values ranged from 4.56 to 4.97, indicating that all study sites were acidic. The soil moisture contents followed the sequence order of EBF>CF>SDF>AM, suggesting that soil moisture content decreased with increasing altitude. The average concentrations of the TOC, TN, TP and TK in the EBF soil were 141.19, 0.75, 0.32 and 24.76 g·kg^-1^, respectively, which were 105.63%, 56.25%, 166.67% and 92.39% higher than those in the AM soil, respectively.

**Table 4:** Soil physical-chemical properties for different vegetation types (mean ±SD).

Vegetation types	pH	Moisture content/ (%)	TOC/ (g·kg^-1^)	TN/ (g·kg^-1^)	TP/ (g·kg^-1^)	TK/ (mg·kg^-1^)
EBF	4.63±0.03c	40.5±1.32a	141.19±1.72a	0.75±0.01a	0.32±0.01a	24.76±0.14a
CF	4.56±0.07bc	38.7±1.26a	136.88±1.13b	0.69±0.01b	0.26±0.01b	21.45±0.11b
SDF	4.83±0.06b	34.9±0.79b	93.17±1.87c	0.55±0.01c	0.15±0.01c	17.65±0.10c
AM	4.97±0.04a	32.1±0.38c	68.66±0.44d	0.48±0.01d	0.12±0.01c	12.87±0.09d

Note: in column followed by the same letter are not significantly different at *P*<0.05 in this and subsequent figures.

Soil nutrients are important carbon and nitrogen sources for soil microorganisms, especially the soil organic matter. To explore the relationship between soil nutrient and the diversity of soil microbial communities, a correlation analysis between soil physical-chemical properties and diversity of microbial community were conducted (Table 5). The results showed a negative correlation relationship between the diversity index of soil microbial communities and soil pH. However, the soil moisture content, TOC, TN, TP and TK were positively correlated with the diversity indexes. Among them, the relationship between diversity index and SOC, TN and TP reached a significant level, indicating that soil nutrient contents play an important role in the determination of soil microbial diversity.

**Table 5:** Correlation analysis between bacterial communities diversity and soil physicochemical property.

Factor	Species Richness (*S*)	Shannon-Weiner index (*H*)	Pielou’s evenness index (*E*)	Simpson index (*D*)
pH	-0.289	-0.421	-0.388	-0.337
moisture content	0.430	0.554	0.581	0.701
TOC	0.986**	0.949*	0.975**	0.990**
TN	0.961**	0.866*	0.963**	0.914*
TP	0.902*	0.858*	0.896*	0.884*
TK	0.794	0.616	0.918*	0.853

## Discussion

Previous studies indicated that the soil bacterial communities were normally comprised of nine major bacterial phyla: *Proteobacteria*, *Actinobacteria*, *Acidobacteria*, *Chloroflexi*, *Bacteroidetes*, *Firmicutes*, *Planctomycetes*, *Verrucomicrobia* and *Gemmatimonadetes* [15]. We observed a total of fourteen bacterial phyla in this study, including *Proteobacteria*, *Bacteroidetes*, *Fusobacterium*, *Actinobacteria*, *Cellulophaga*, *Arthrobacter*, *Lactobacillus*, *Clostridium*, *Mycoplasma*, *Nitrospira*, *Streptococcus*, *Desulfobacter*, *Staphylococcus* and *Chloroflexi*. Similar results were also reported in other mountains [27]. In the present study, *Actinobacteria*, *Fusobacterium*, *Bacteroidetes* and *Proteobacteria* were the most predominant phyla in all the soil samples.

The diversity of soil bacterial communities decreased with increasing elevation, and showed a general trend of EFB>CF>SDF>AM. A correlation analysis of microbial diversity and soil physicochemical properties also indicates that the soil nutrient indicators such as TOC, TN, TP and TK tend to decrease with an increment in altitude. Our results demonstrated close links between soil nutrient contents and bacterial diversity, and the correlation coefficients between the four diversity indexes and TOC or TN were all greater than 0.86 (*p* <0.05). Therefore, the most important reason for the difference in soil microbial diversity along an altitude gradient is the decline of soil nutrient content. Furthermore, the soil tiny animals, plant root and seasonal variation might also cause or contribute to the differences [28]. The effect of TOC on the compositions of bacterial communities has been reported previously in other research sites [23, 24]. TOC concentration may have a direct effect on the bacterial composition or through the changes in biomass and composition of forest soil. Our field investigation found that EBF, located in lowest altitude, had most abundant biological diversity and forest litter, whereas the degree of biological diversity and forest litter decreased with an increase in altitude in CF and SDF. The AM site contains the lowest biodiversity. The decline of vegetation diversity and forest litter inevitably affect forest soil physical and chemical properties, especially the TOC content, thereby reduce the abundance of soil microorganisms [29]. Furthermore, the effects of spatial change on ecosystem are also very important [30–32]. The similar researches in different study sites may get the opposite conclusion. We will do further studies to compare the variation among different forest ecosystems in subtropical area.

In conclusion, the present study shows remarkable differences in the bacterial communities’ composition under different vegetation types of the Wuyi Mountains. Multivariate statistical analysis indicates that TOC has a significant effect on the structure of bacterial communities in all soil samples. However, the T-RFLP method we used has limitation to detect characteristics of soil microbial community. Future work will be performed by combing with other soil microbial research methods such as denaturing gradient gel electrophoresis (DGGE) and phospholipid fatty acid (PLFA). This study also reveals the interactions between bacterial communities’ composition and certain soil characteristics, and the roles of microorganisms involved in the biogeochemical cycling of nutrient elements in this forest ecosystem.

## Supporting Information

S1 File The experimental data of T-RFLP. The data includes five tables and one figure as follows. T-RFLP database for four enzyme digestions (**Table A**). All T-RFs detected using T-RFLP (**Table B**). Bacterial species detected by Hae III (**Table C**). Bacterial community detected in all soil samples (**Table D**). Principal components analysis (**Table E**). T-RFLP profiles digested by four restriction enzymes in rhizosphere soil (**Fig. A**).
(ZIP)
