# Supplementary material for: Terminal Restriction Fragment Length Polymorphism Analysis of Soil Bacterial Communities under Different Vegetation Types in Subtropical Area
Source: PLoS One. 2015 Jun 22;10(6):e0129397. doi: 10.1371/journal.pone.0129397 (PMC4476674; doi:10.1371/journal.pone.0129397)

Sample File	Sample Name	Panel	SQO	OS	SQ
2013-12-02_111_A01.fsa	111	None			

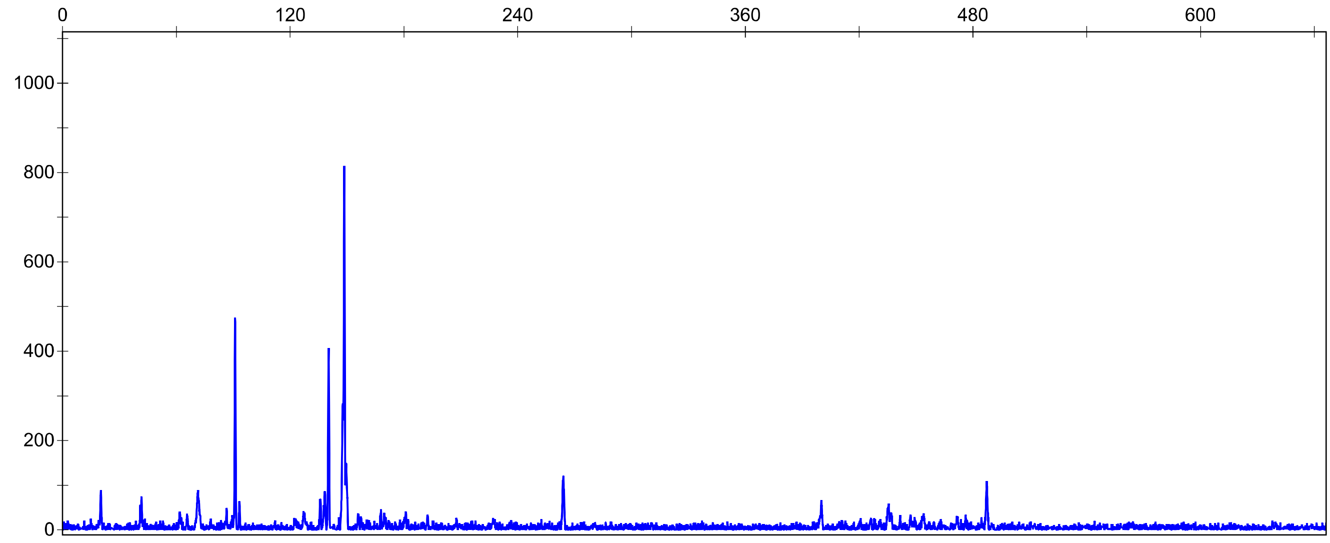

Sample File	Sample Name	Panel	SQO	OS	SQ
2013-12-02_121_B01.fsa	121	None			

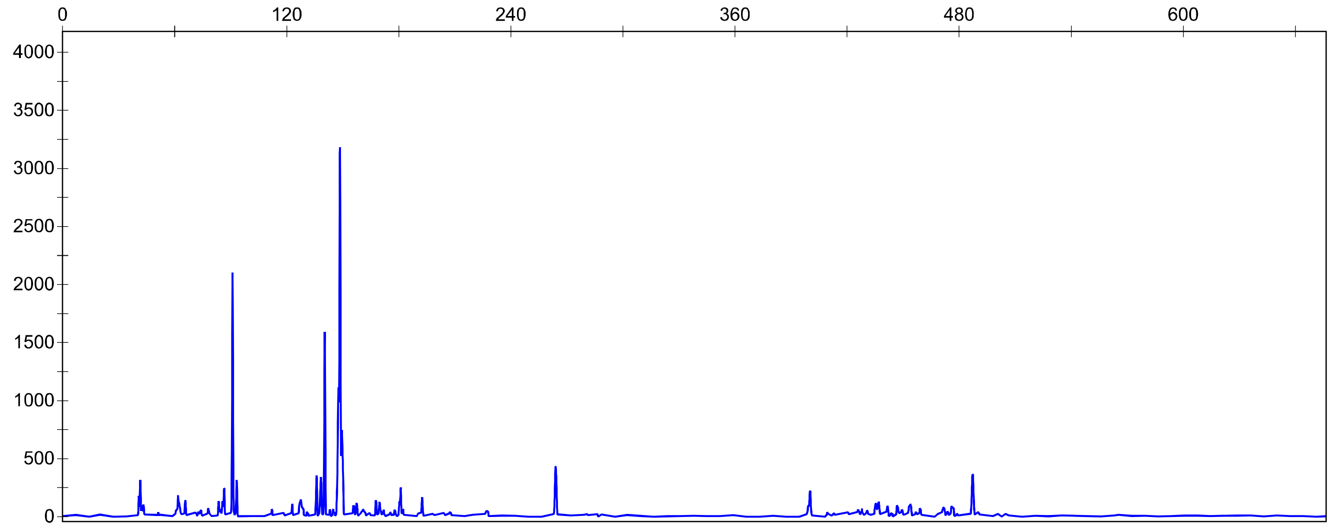

Sample File	Sample Name	Panel	SQO	OS	SQ
2013-12-02_131_C01.fsa	131	None			

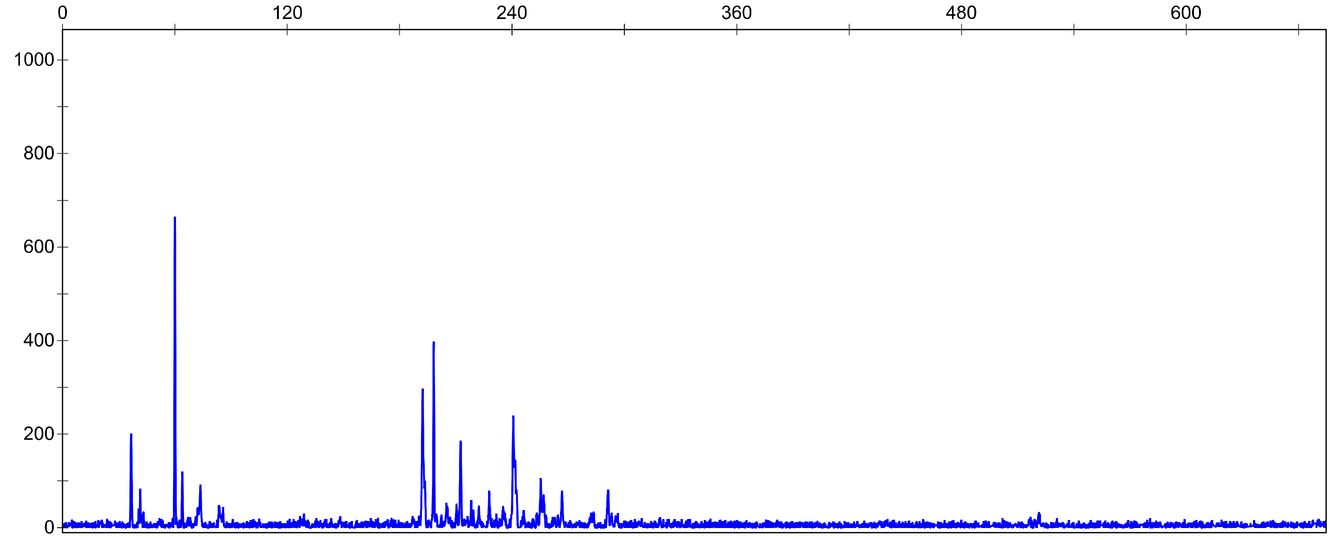

Sample File	Sample Name	Panel	SQO	OS	SQ
2013-12-02_141_D01.fsa	141	None			

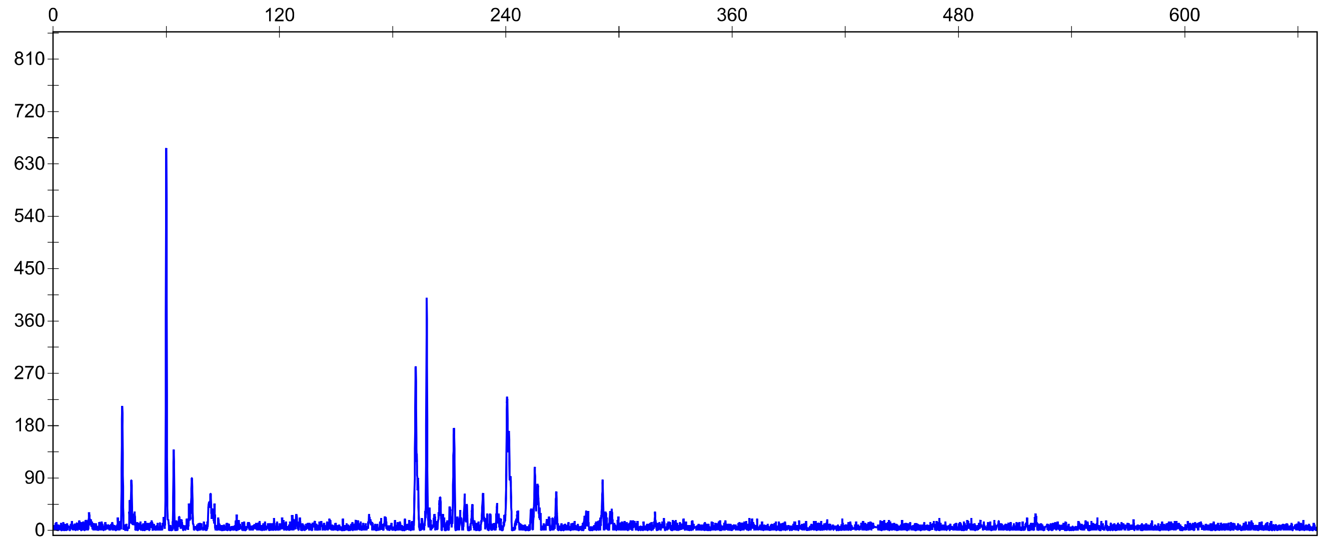

2013-12-02_151_E01.fsa	151	None			

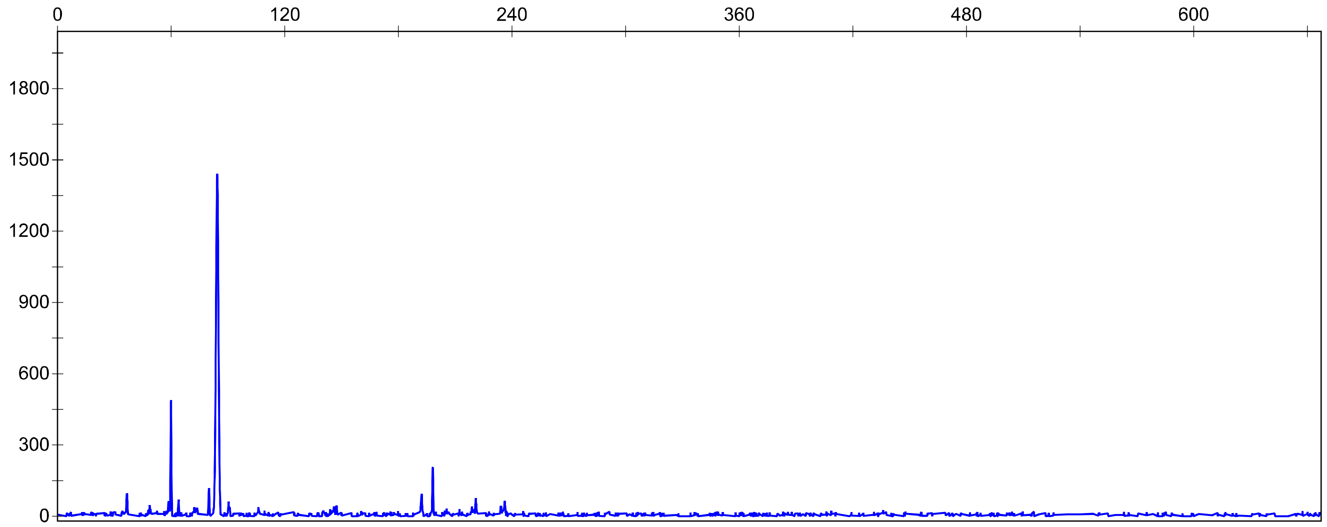

2013-12-02_161_F01.fsa	161	None			

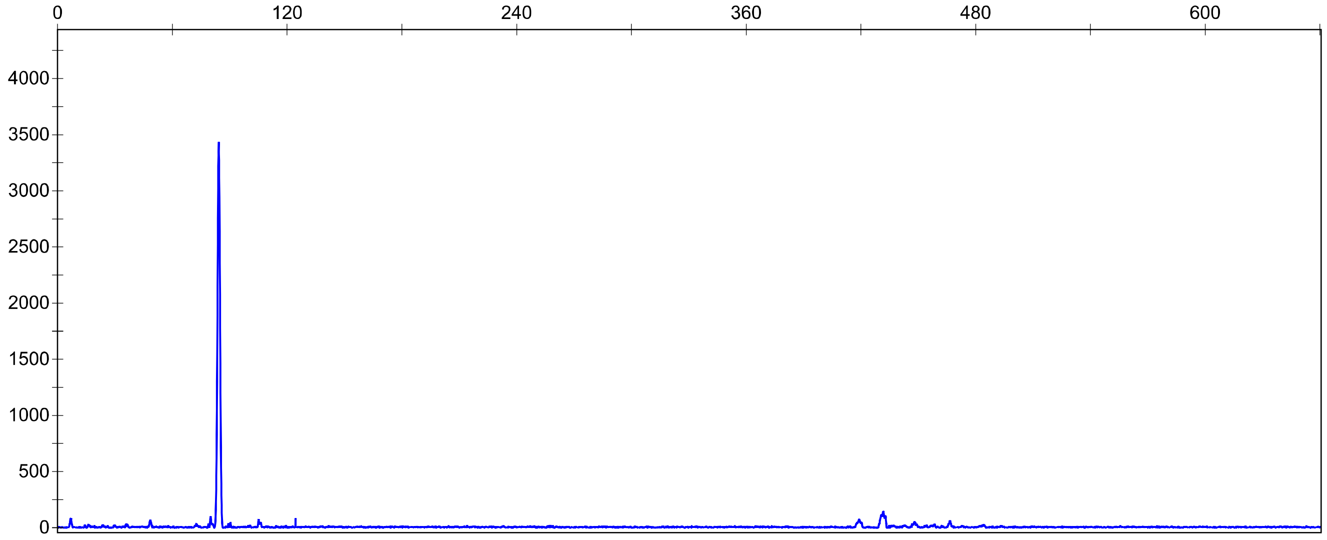

Sample File	Sample Name	Panel	SQO	OS	SQ
2013-12-02_171_G01.fsa	171	None			

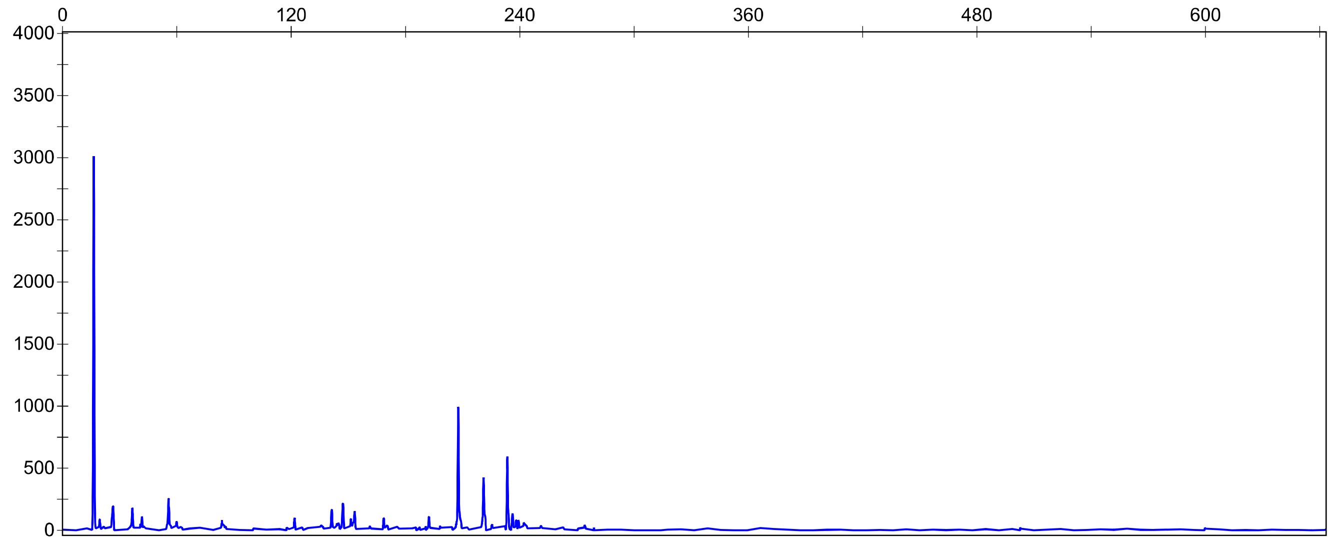

2013-12-02_181_H01.fsa	181	None			

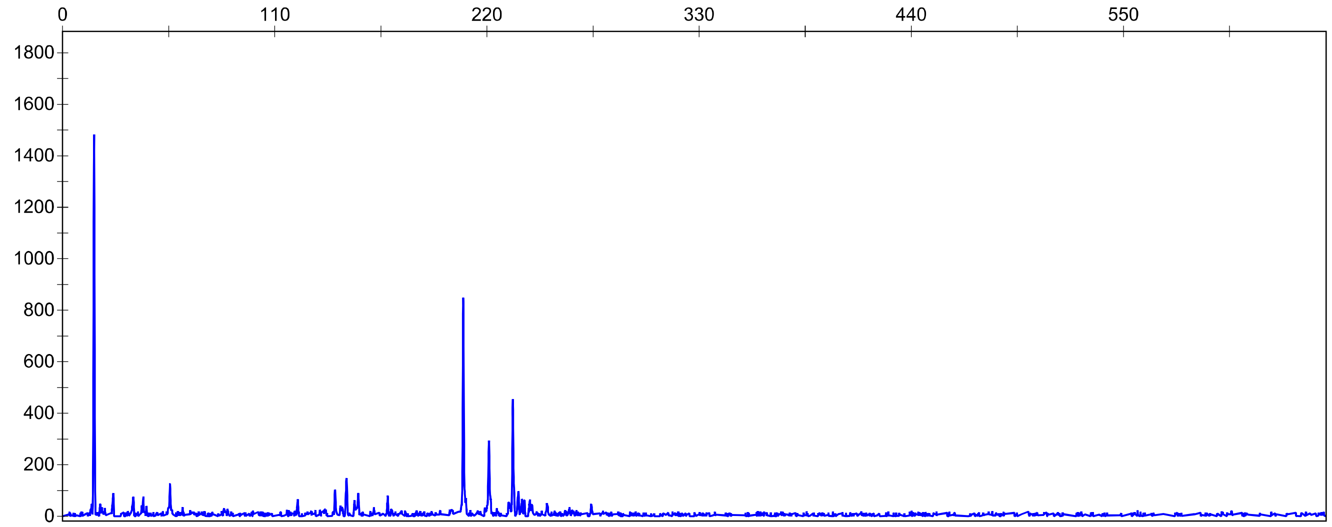

2013-12-02_211_A02.fsa	211	None			

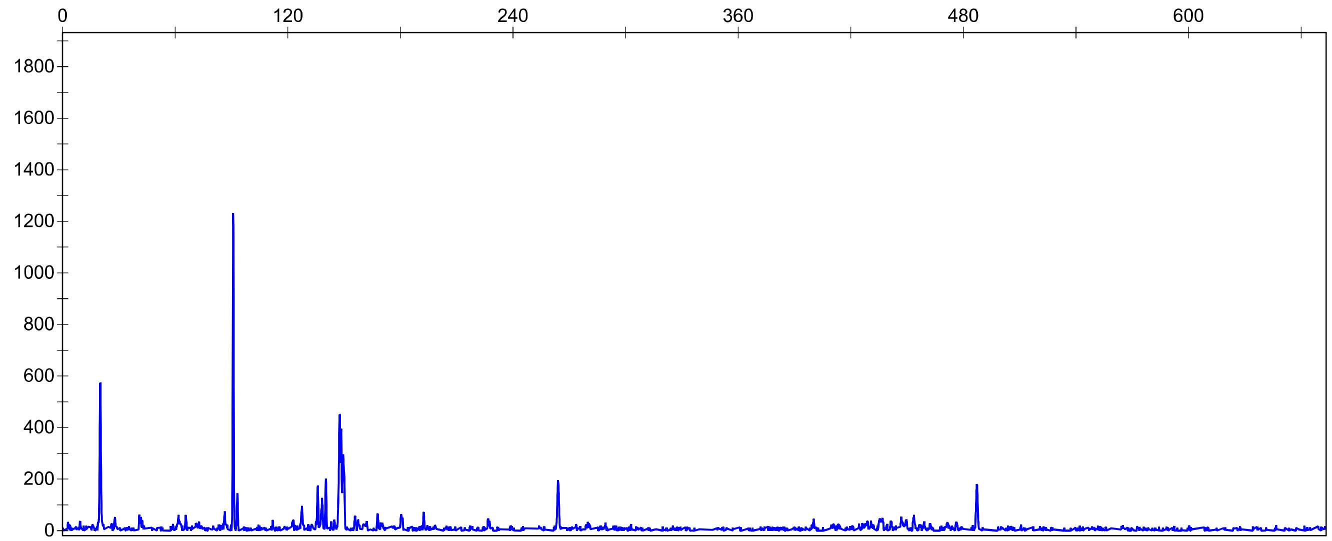

Sample File	Sample Name	Panel	SQO	OS	SQ
2013-12-02_221_B02.fsa	221	None			

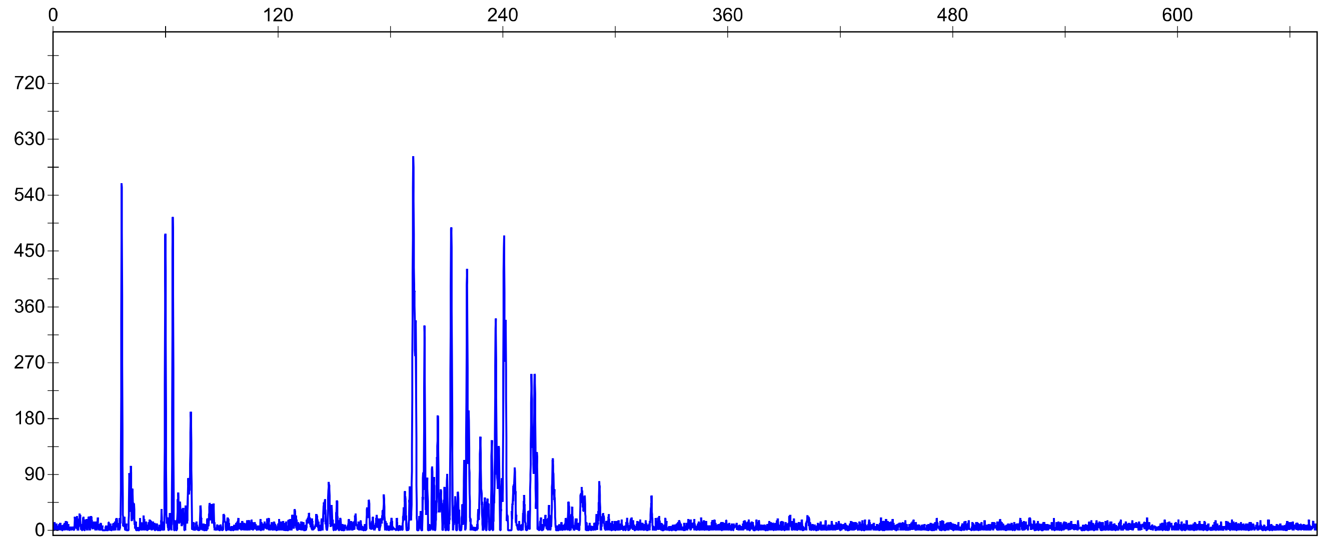

2013-12-02_231_C02.fsa	231	None			

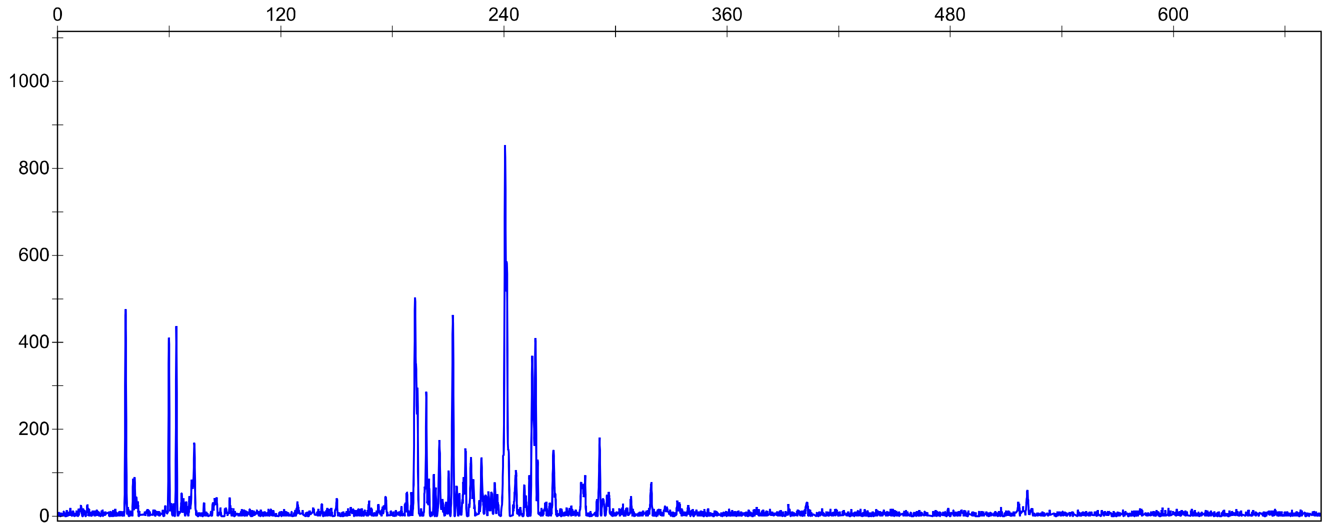

2013-12-02_241_D02.fsa	241	None			

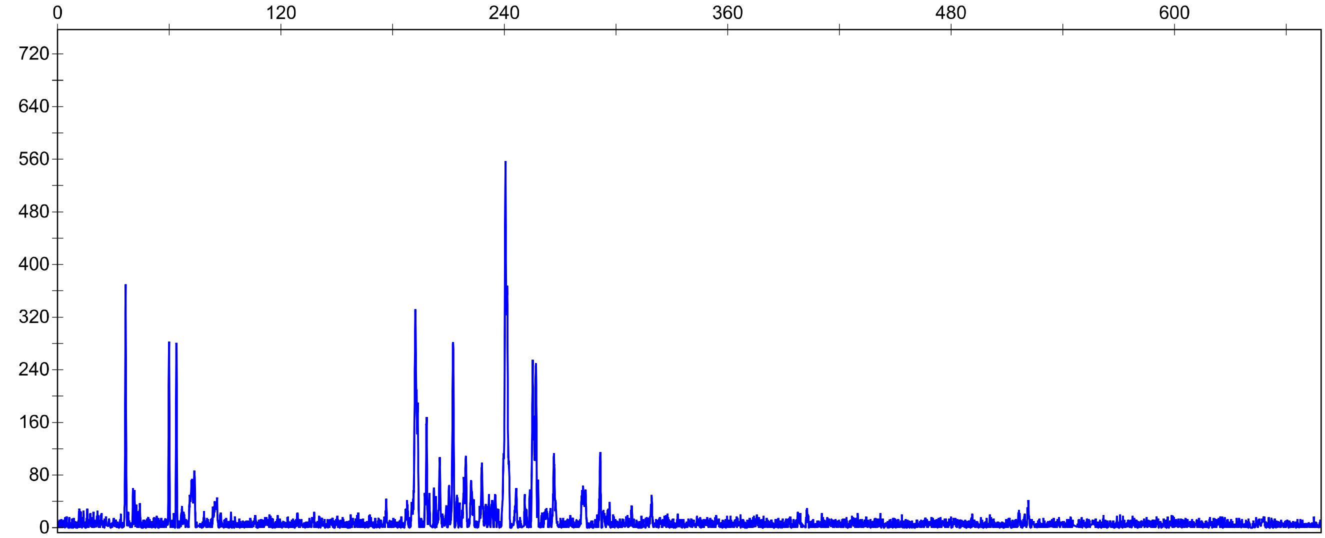

Sample File	Sample Name	Panel	SQO	OS	SQ
2013-12-02_251_E02.fsa	251	None			

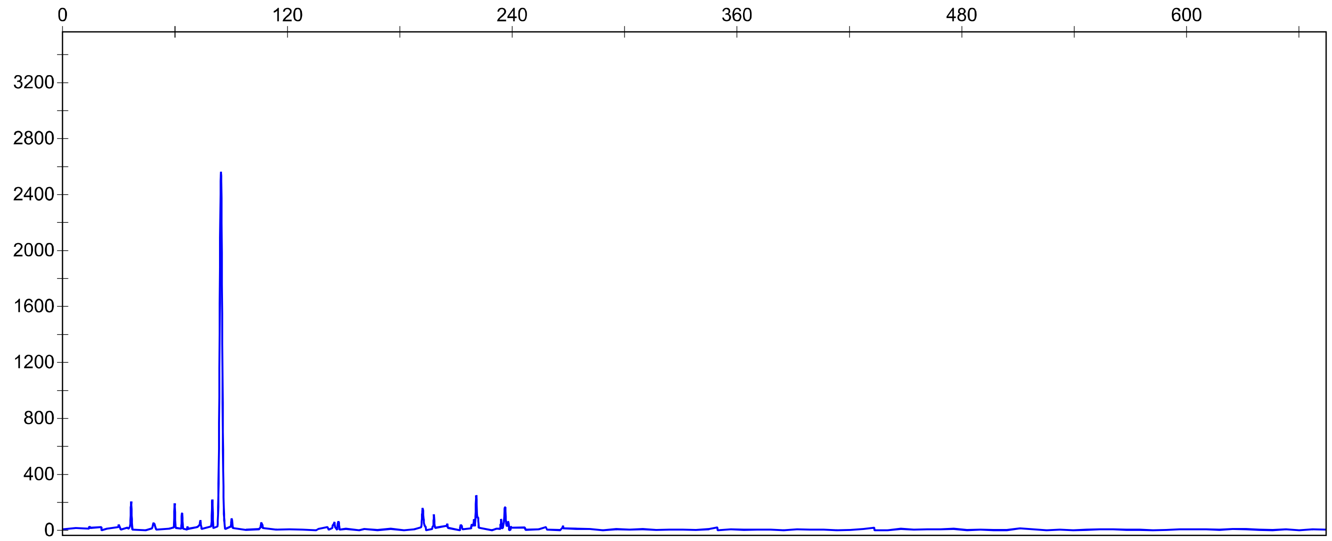

2013-12-02_261_F02.fsa	261	None			

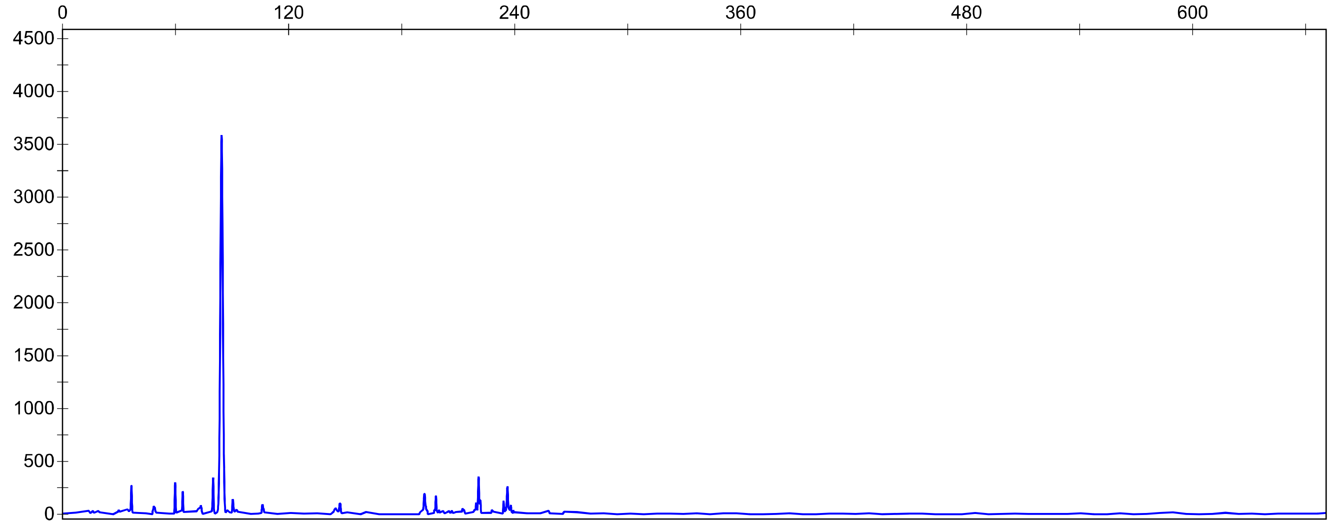

2013-12-02_271_G02.fsa	271	None			

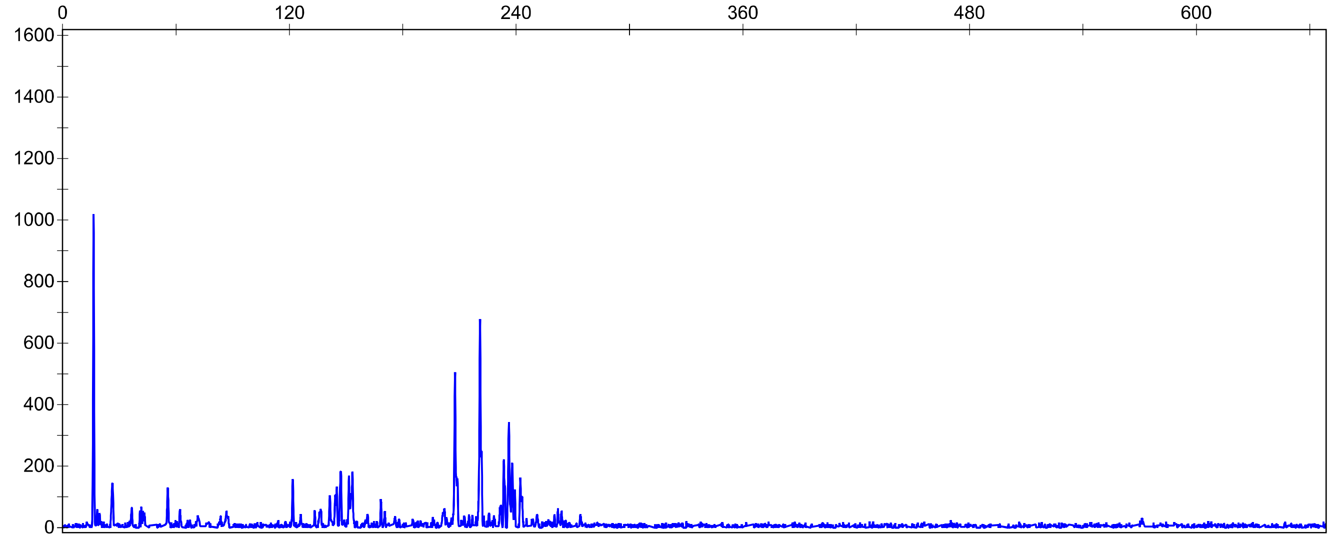

Sample File	Sample Name	Panel	SQO	OS	SQ
2013-12-02_281_H02.fsa	281	None			

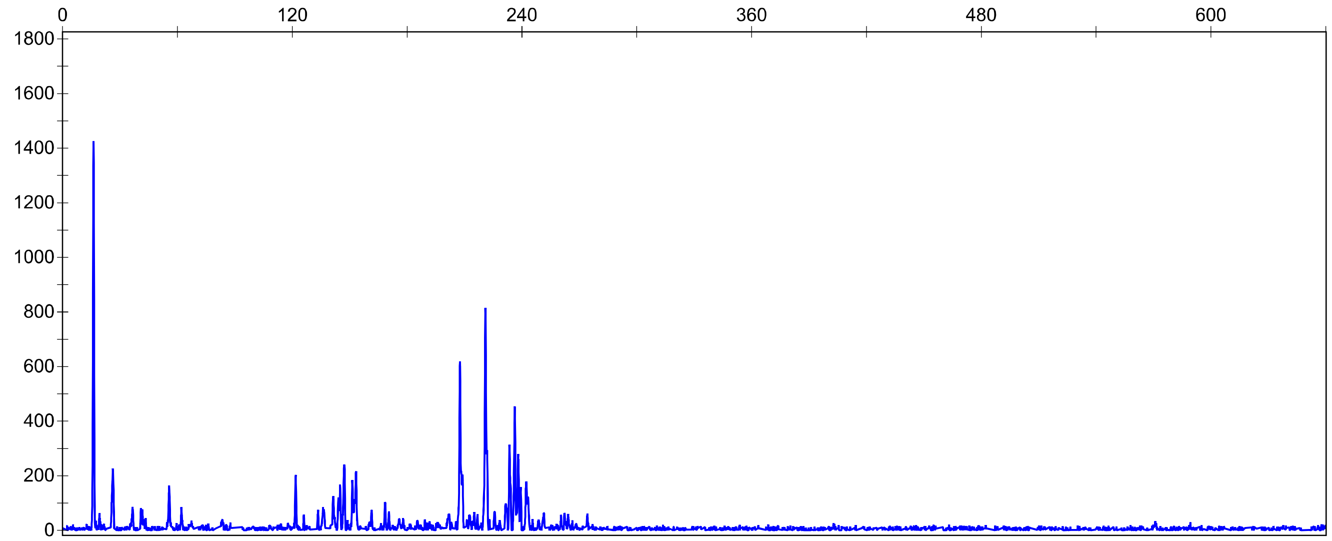

Sample File	Sample Name	Panel	SQO	OS	SQ
2013-12-02_311_A03.fsa	311	None			

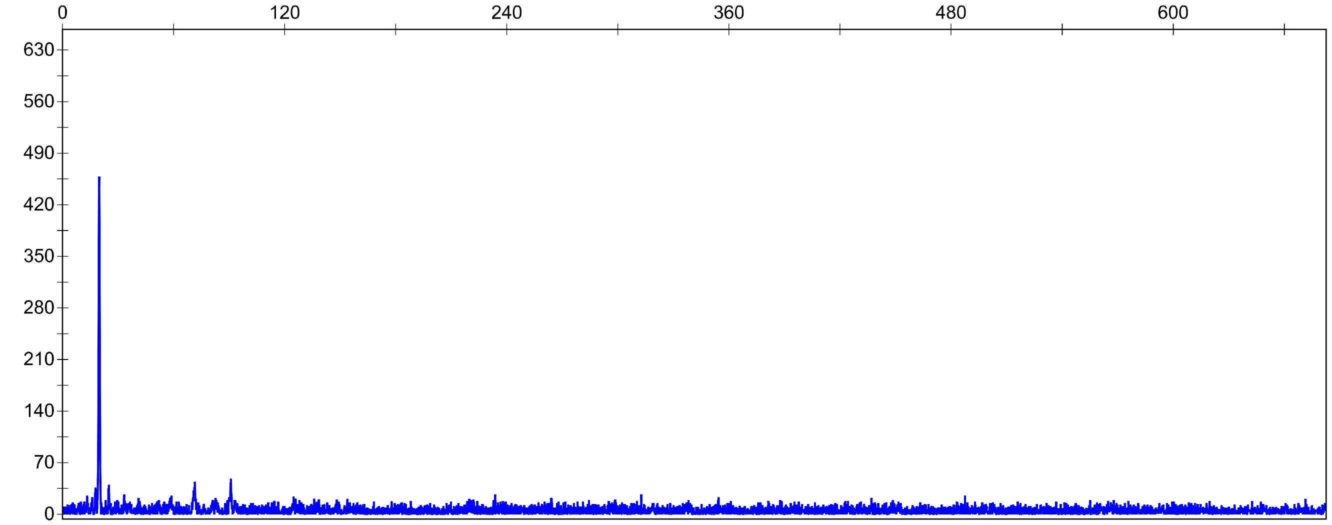

Sample File	Sample Name	Panel	SQO	OS	SQ
2013-12-02_321_B03.fsa	321	None			

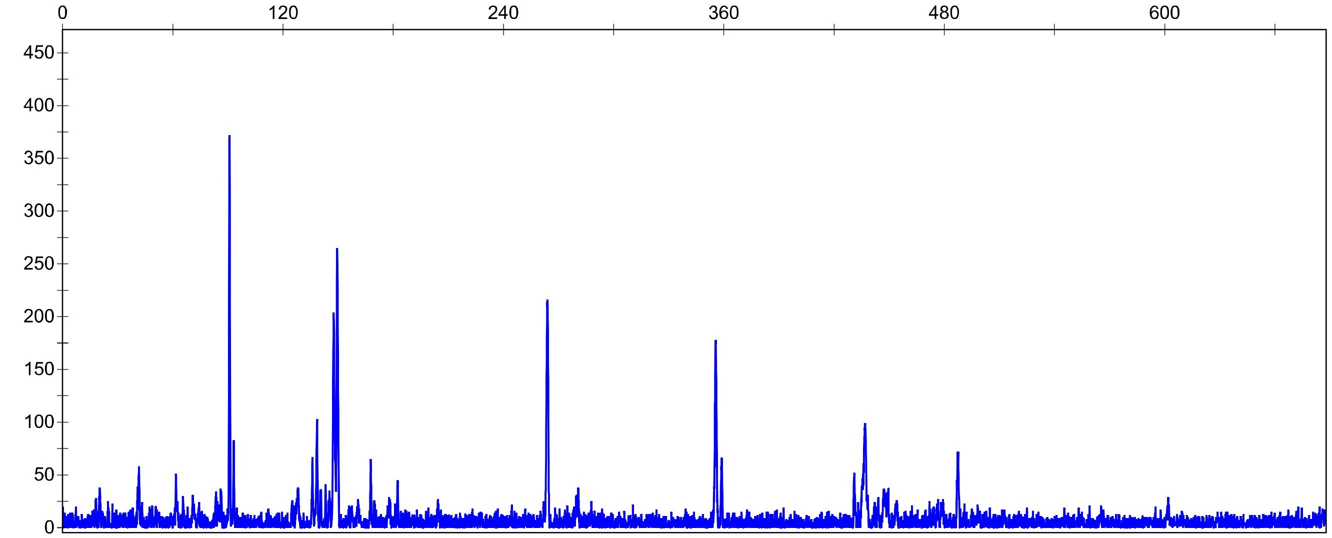

Sample File	Sample Name	Panel	SQO	OS	SQ
2013-12-02_331_C03.fsa	331	None			

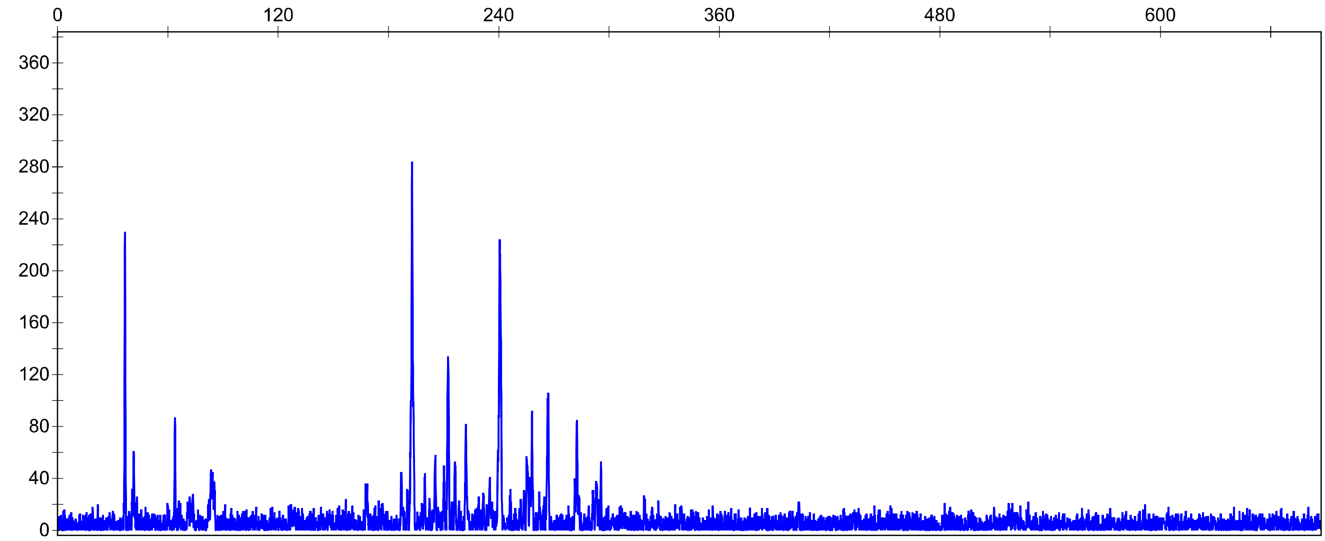

Sample File	Sample Name	Panel	SQO	OS	SQ
2013-12-02_341_D03.fsa	341	None			

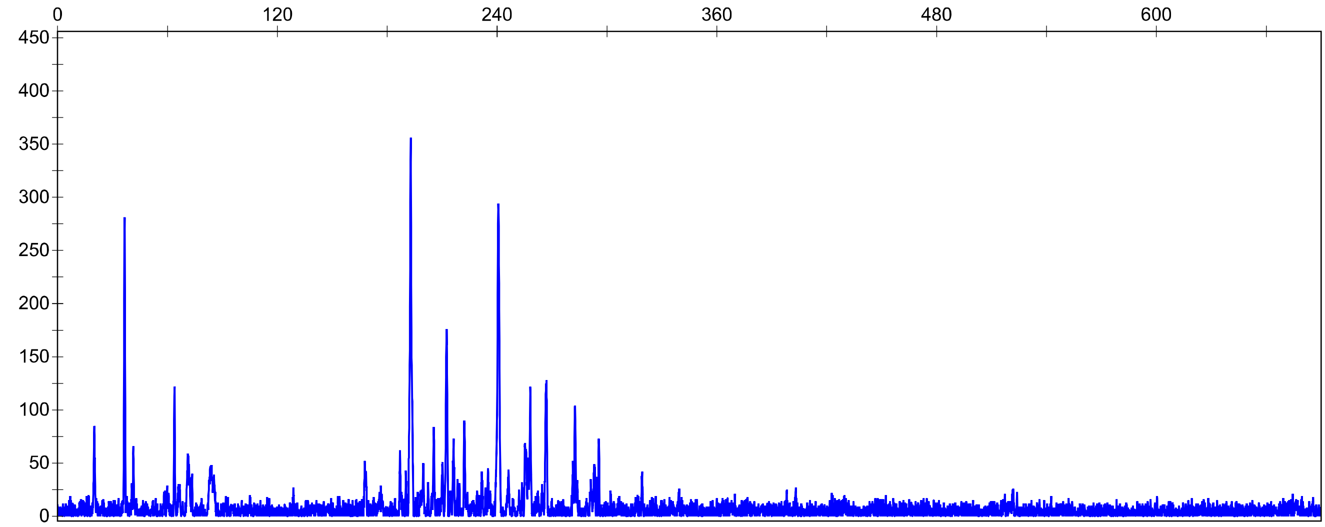

Sample File	Sample Name	Panel	SQO	OS	SQ
2013-12-02_351_E03.fsa	351	None			

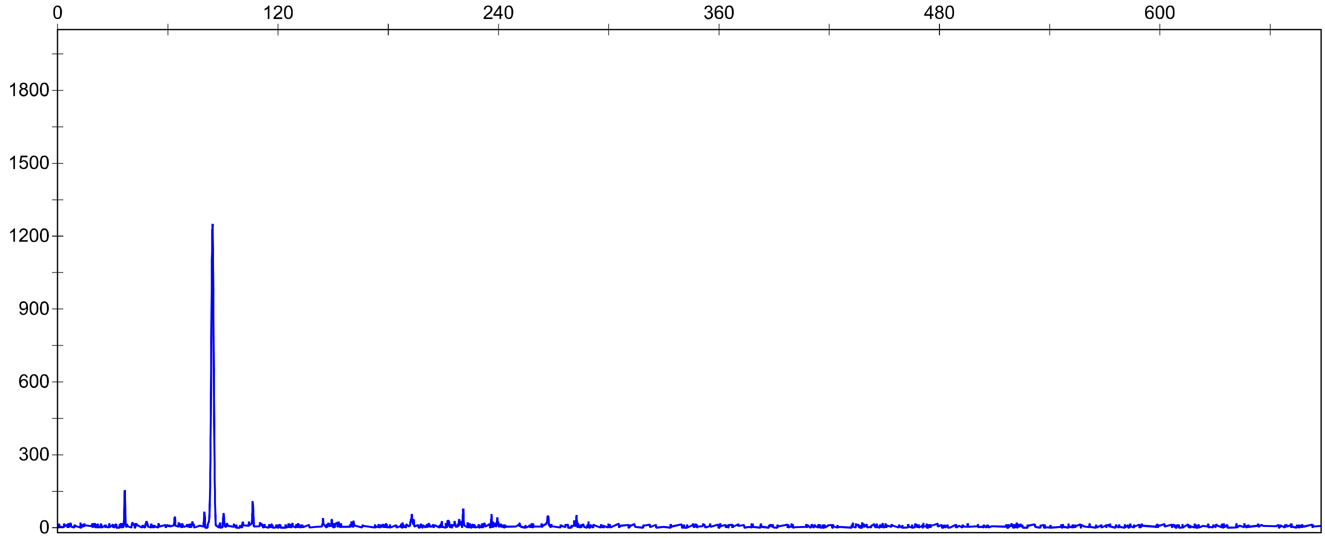

Sample File	Sample Name	Panel	SQO	OS	SQ
2013-12-02_361_F03.fsa	361	None			

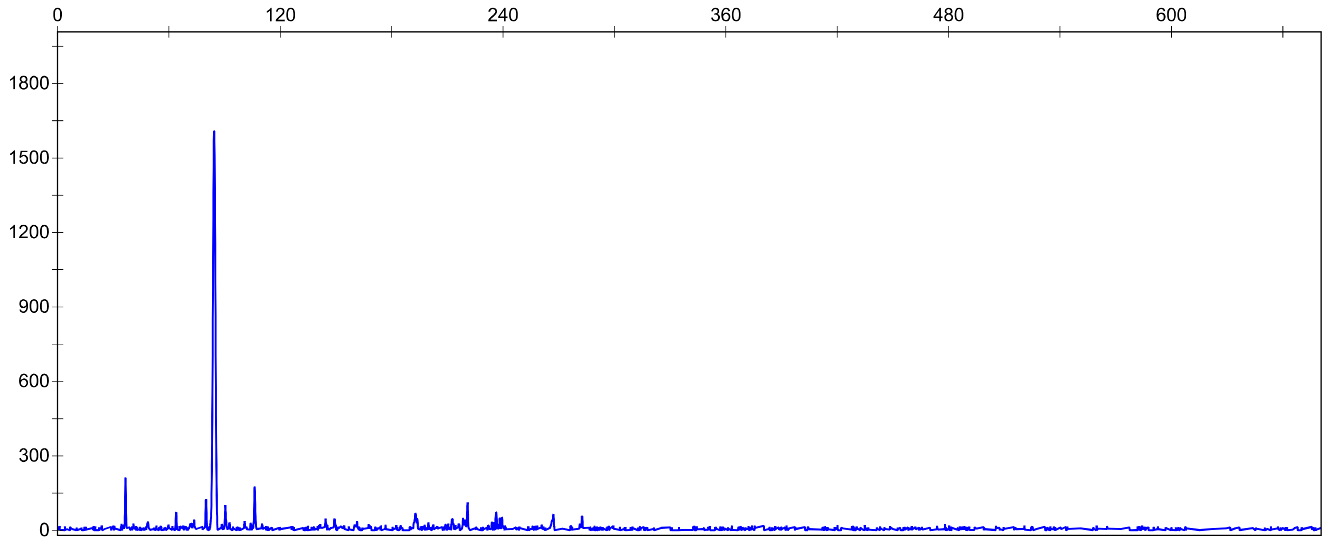

Sample File	Sample Name	Panel	SQO	OS	SQ
2013-12-02_371_G03.fsa	371	None			

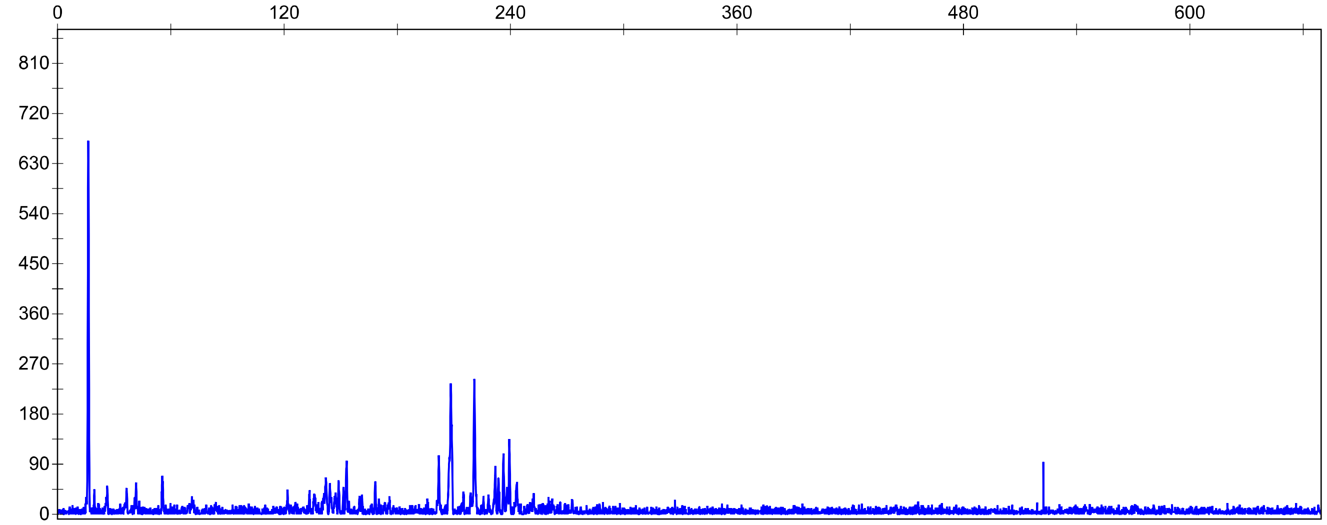

Sample File	Sample Name	Panel	SQO	OS	SQ
2013-12-02_381_H03.fsa	381	None			

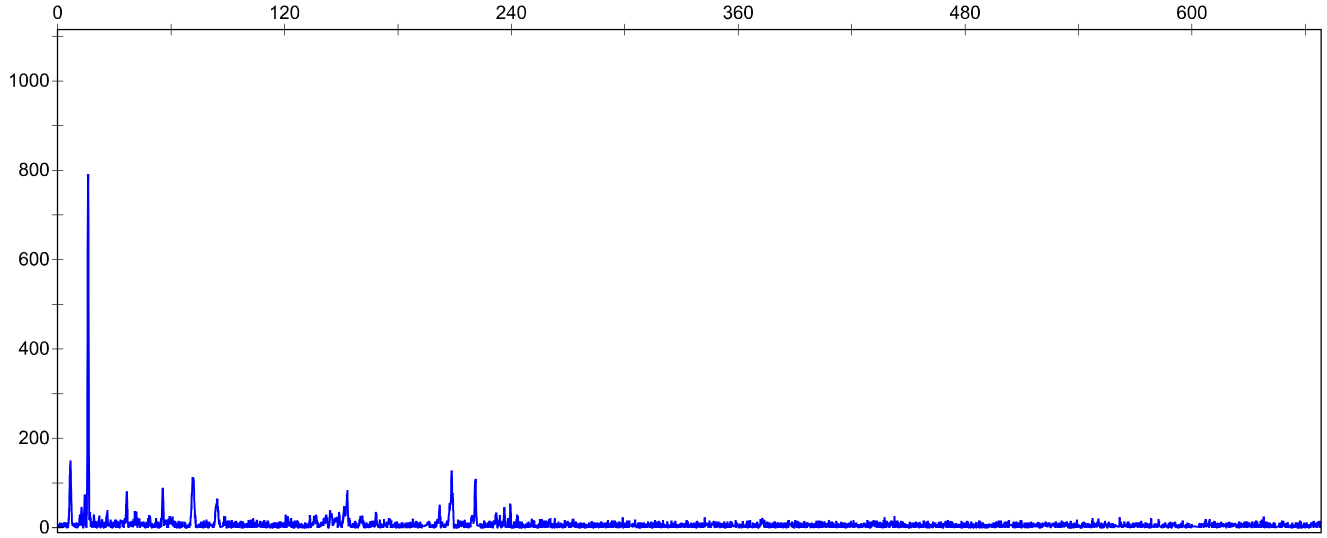

Sample File	Sample Name	Panel	SQO	OS	SQ
2013-12-02_411_A04.fsa	411	None			

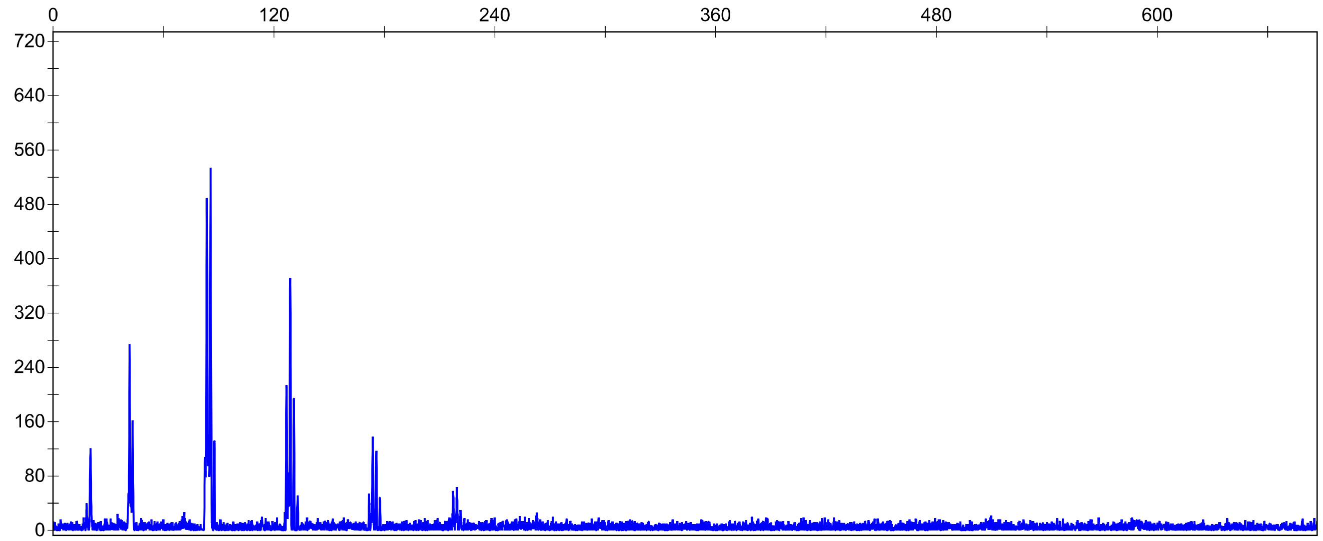

2013-12-02_421_B04.fsa	421	None			

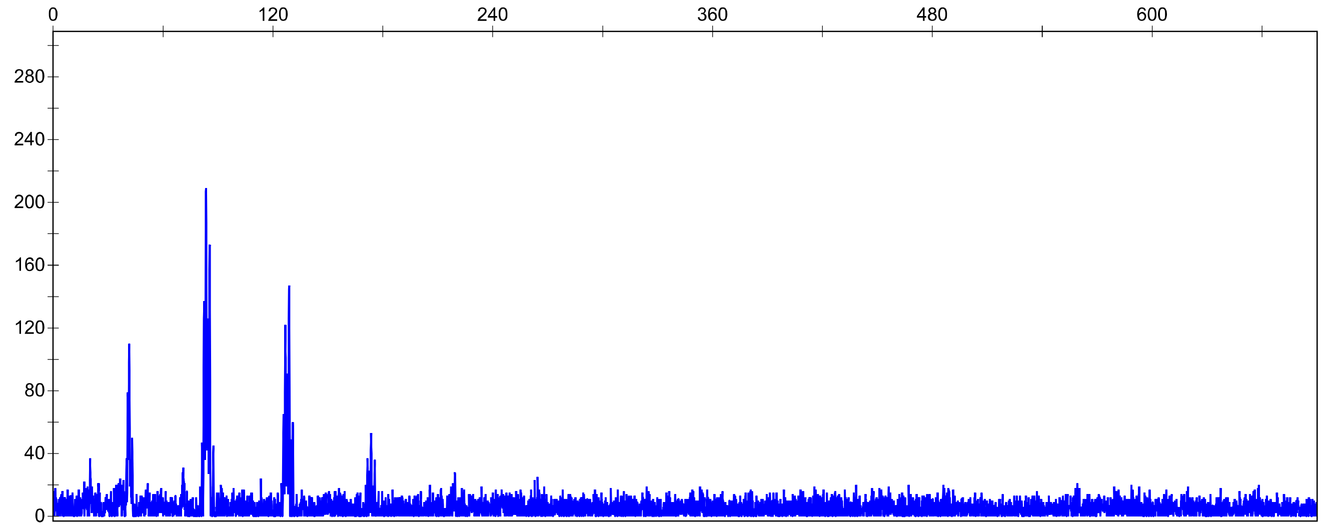

2013-12-02_431_C04.fsa	431	None			

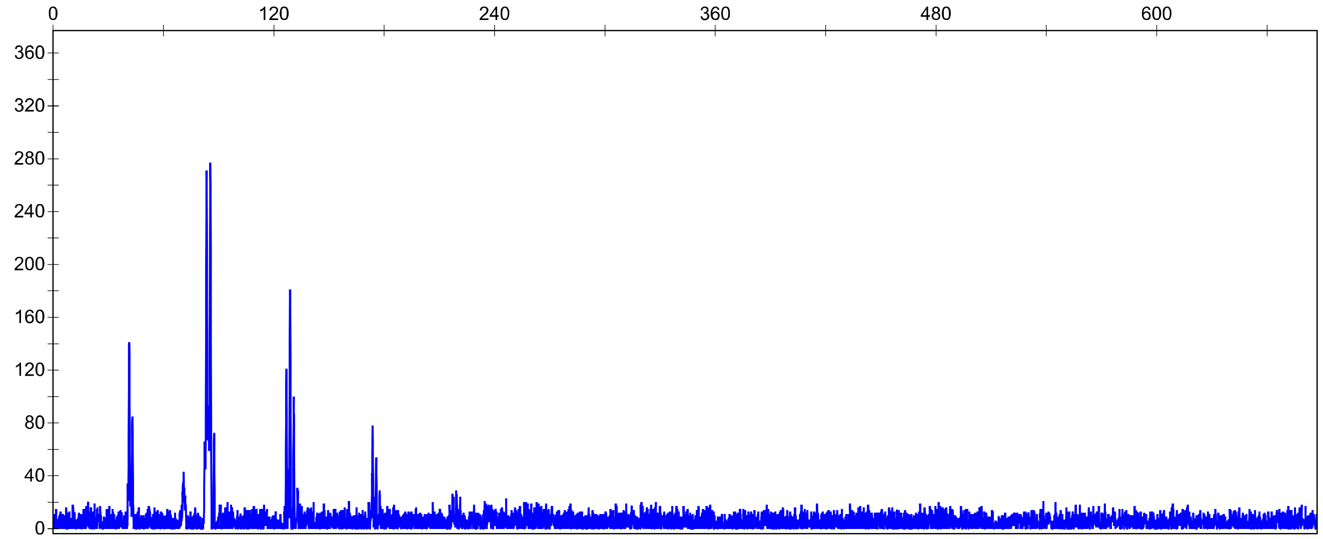

Sample File	Sample Name	Panel	SQO	OS	SQ
2013-12-02_441_D04.fsa	441	None			

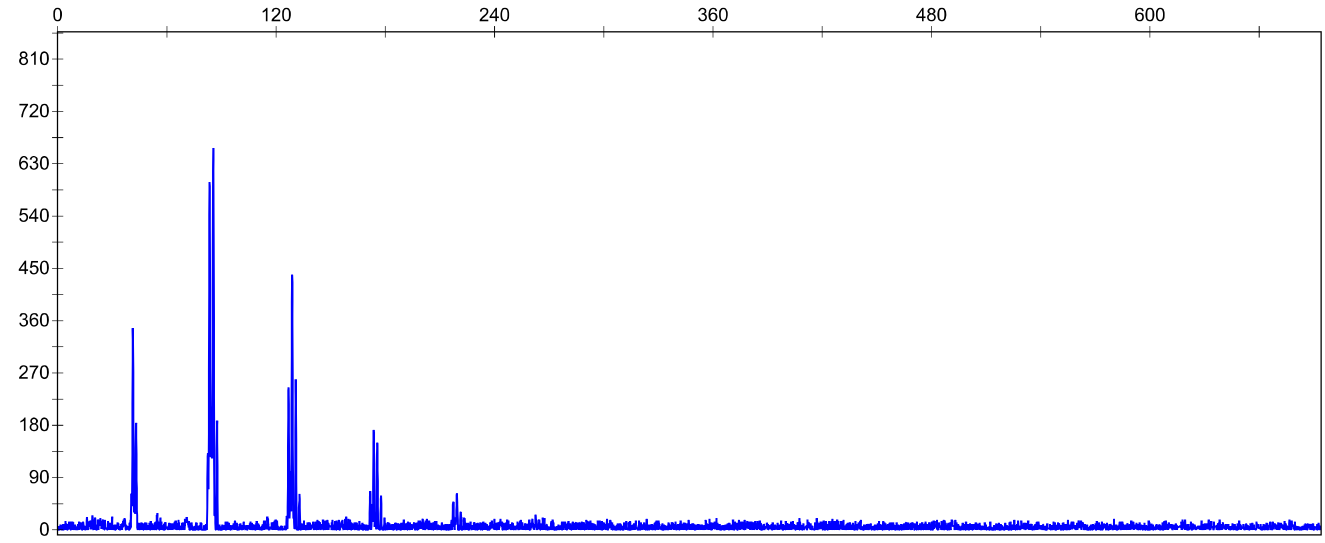

Sample File	Sample Name	Panel	SQO	OS	SQ
2013-12-02_451_E04.fsa	451	None			

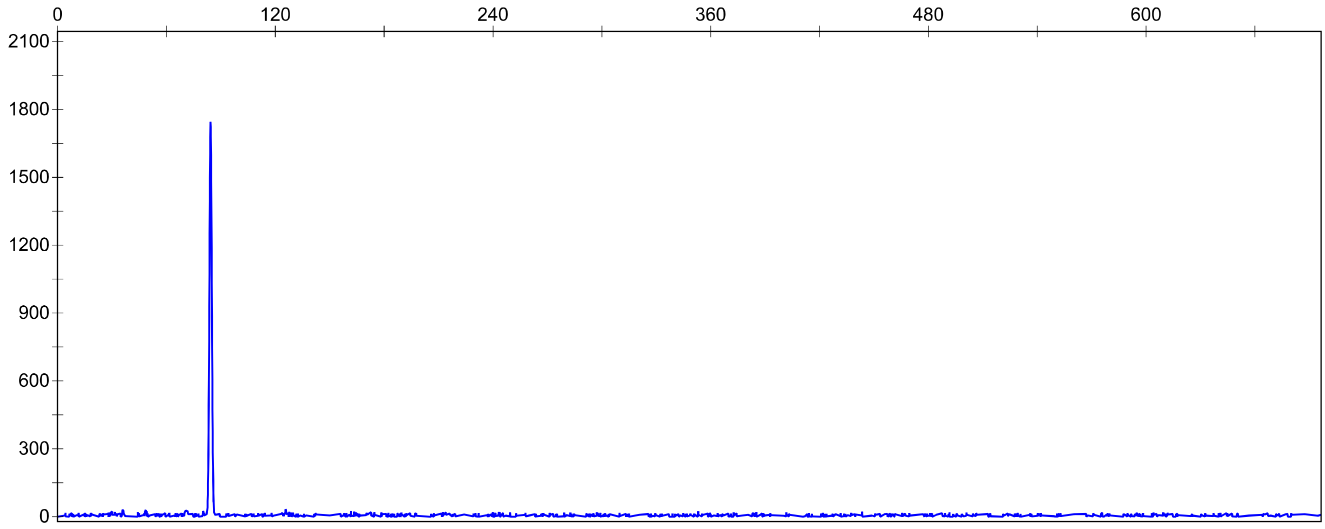

Sample File	Sample Name	Panel	SQO	OS	SQ
2013-12-02_461_F04.fsa	461	None			

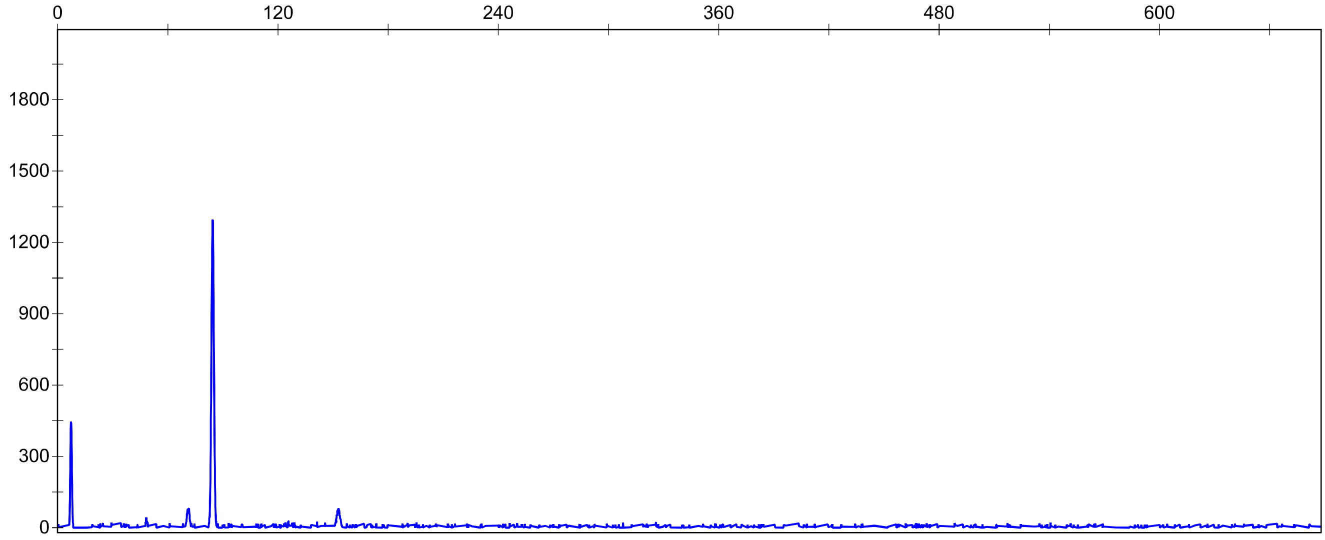

Sample File	Sample Name	Panel	SQO	OS	SQ
2013-12-02_471_G04.fsa	471	None			

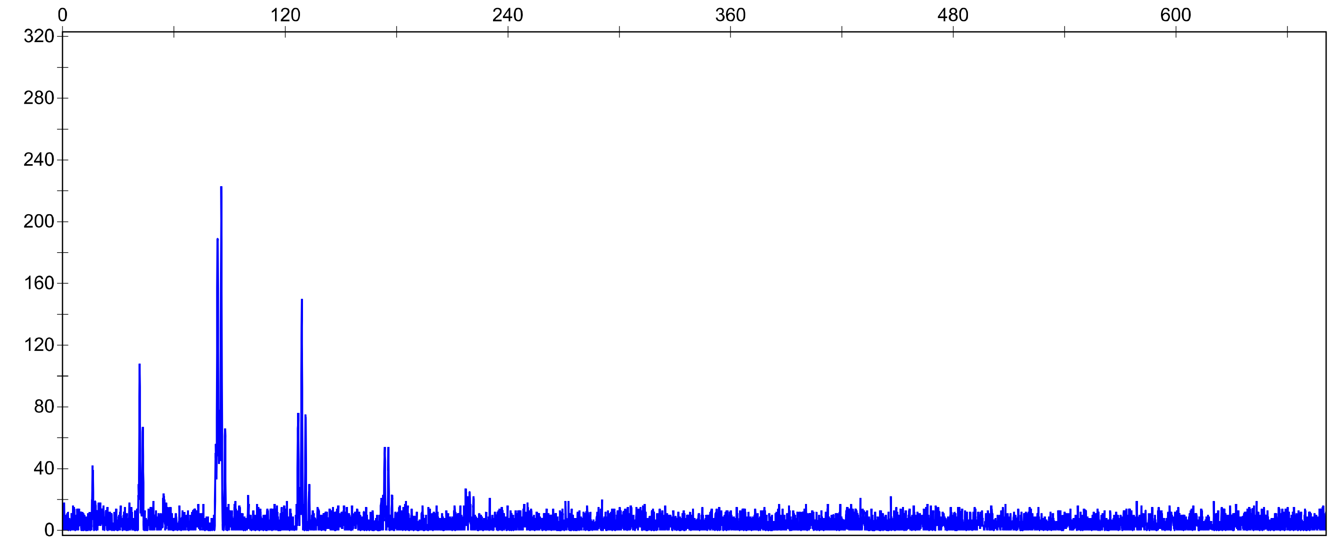

2013-12-02_481_H04.fsa	481	None			

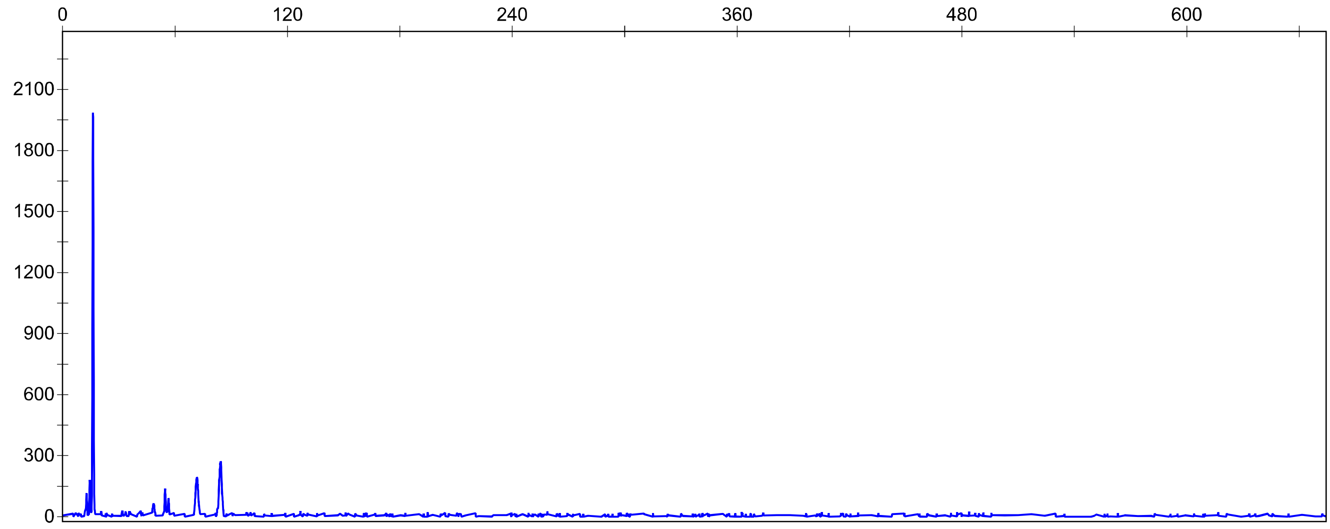

Supplement: S1 File — The data includes five tables and one figure as follows. T-RFLP database for four enzyme digestions (Table A). All T-RFs detected using T-RFLP (Table B). Bacterial species detected by Hae III (Table C). Bacterial community detected in all soil samples (Table D). Principal components analysis (Table E). T-RFLP profiles digested by four restriction enzymes in rhizosphere soil (Fig. A). (ZIP) [file pone.0129397.s001.zip › Supporting Information/Fig. A T-RFLP profiles digested by four restriction enzymes in rhizosphere soil.pdf]
